# The neuro–metabolic–immune axis in breast cancer: emerging mechanisms and subtype-specific therapeutic opportunities

**DOI:** 10.3389/fimmu.2026.1890810

**Published:** 2026-10-19

**Authors:** Nannan Xia, Renhong Huang, Hanyi Zhong, Ziling Zhou, Shoutang Wang, Zheng Wang, Kunwei Shen

**Affiliations:** 1 Department of General Surgery, Comprehensive Breast Health Center, Ruijin Hospital, Shanghai Jiao Tong University School of Medicine, Shanghai, China; 2 School of Biomedical Sciences, Li Ka Shing Faculty of Medicine, University of Hong Kong, Pok Fu Lam, Hong Kong, China

**Keywords:** breast cancer, metabolic reprogramming, neuro-metabolic-immune axis, tumor innervation, tumor microenvironment

## Abstract

Tumor innervation is increasingly recognized as an active component of the breast tumor microenvironment, where neural signals influence cancer cells, stromal populations, and immune responses through neurotransmitters, neuropeptides, and neurotrophic factors. Sympathetic, cholinergic, and sensory pathways can regulate tumor growth, migration, stromal remodeling, and immune activity, while neural signaling also intersects with tumor metabolism by modulating glycolysis, lipid mobilization, and amino acid utilization. Metabolic changes, including lactate accumulation and histone lactylation, may further reshape tumor and immune-cell states, linking neural activity with metabolic adaptation and immune regulation. These interactions vary across breast cancer subtypes, with adrenergic and sensory-neuropeptide signaling implicated in triple-negative breast cancer, metabolic adaptation during HER2-targeted therapy, and neurotrophic and adipose-tumor crosstalk in hormone receptor-positive/HER2-negative disease. We apply the recently proposed concept of the neuro–metabolic–immune axis to breast cancer, systematically evaluating the strength of evidence for each component and organizing the discussion by molecular subtype and therapeutic maturity. We further discuss therapeutic opportunities ranging from β-adrenergic and neuropeptide pathway modulation to downstream metabolic targeting, while distinguishing direct neural mechanisms from indirect or complementary evidence.

## 1 Introduction

Breast cancer remains one of the most common malignancies worldwide, and a major cause of cancer-related morbidity and mortality in women (1–4). Although advances in molecular subtyping, targeted therapy, and immunotherapy have substantially improved patient outcomes, metastasis, therapeutic resistance, and disease recurrence remain major clinical challenges. The tumor microenvironment (TME) is a dynamic ecosystem composed of cancer cells, immune and stromal populations, vascular components, extracellular matrix, and diverse signaling molecules (5). Growing evidence indicates that neural components also participate in the regulation of this ecosystem. Peripheral nerve fibers can infiltrate or surround tumor tissues and release neurotransmitters and neuropeptides that act on cancer cells, stromal cells, and immune populations (6–9). In breast cancer, histopathological studies have linked tumor innervation and increased nerve density with higher-grade disease and poorer clinical outcomes (10, 11).

Metabolic reprogramming is another established feature of breast cancer and involves alterations in glucose utilization, lipid metabolism, and amino acid metabolism. These changes support tumor growth and survival while also shaping immune-cell function through nutrient competition and bioactive metabolites (12–14). Breast cancer-specific studies further show that β-adrenergic signaling can modulate glucose metabolism through HK2 and LDHA and can promote lipid mobilization through stress-induced lipolysis (15–17). Neural-metabolic interactions have also been demonstrated in specialized settings such as breast cancer brain metastasis, where metastatic cells exploit neuron-derived glutamatergic signaling and can reprogram local neurometabolic coupling (18, 19). Metabolites generated within the TME can in turn modify immune-cell activity and tumor-cell behavior, creating additional points of convergence between neural and metabolic regulation.

Direct links between neural signaling and tumor metabolism are comparatively less established than neuro-immune or metabolic-immune interactions. Their biological consequences depend on the neural mediator, receptor subtype, responding cell population, breast cancer subtype, and treatment context. A broader neuro-metabolic-immune framework has recently been proposed to integrate neurotransmitter signaling, metabolic reprogramming, and immune evasion across cancer contexts (12). In parallel, previous reviews have synthesized nervous-system interactions specifically in breast cancer and neuro-immune regulation across multiple malignancies (8, 13). Building on these foundations, we apply the neuro-metabolic-immune framework specifically to breast cancer, while treating it as a network of potentially interconnected interfaces rather than a single demonstrated causal axis.

The contribution of the present review is therefore not to introduce the axis itself, but to provide a breast-cancer-specific and evidence-graded synthesis of its constituent mechanisms. We organize the evidence across triple-negative, HER2-positive, and hormone receptor-positive/HER2-negative breast cancer, with particular attention to the cellular and molecular context in which each interaction has been demonstrated. Mechanistically, we distinguish relationships demonstrated directly within breast cancer models from experimentally supported pairwise neural–metabolic or neural–immune interfaces, breast cancer metabolic or immune mechanisms with only indirect neural links, and complementary cross-tumor or associative evidence. Therapeutic strategies are further considered according to their translational maturity, separating established downstream treatments, prospective interventional evidence, retrospective or observational associations, preclinical findings, and hypothesis-generating opportunities.

Accordingly, we first summarize the major neural and neuroactive components implicated in breast cancer, including sympathetic, cholinergic, and sensory signaling, Schwann cells, and additional neuroactive and neural-guidance pathways. We then examine experimentally supported neural interfaces with glucose, lipid, amino acid, and lactate-associated metabolism and their relationships with immune and stromal states. Finally, we evaluate therapeutic implications across breast cancer subtypes, distinguishing interventions directed at neural or neuropeptide pathways from downstream metabolic or immune vulnerabilities and interpreting each strategy according to its current evidence level and clinical maturity (**Figure 1**).

**Figure 1:**
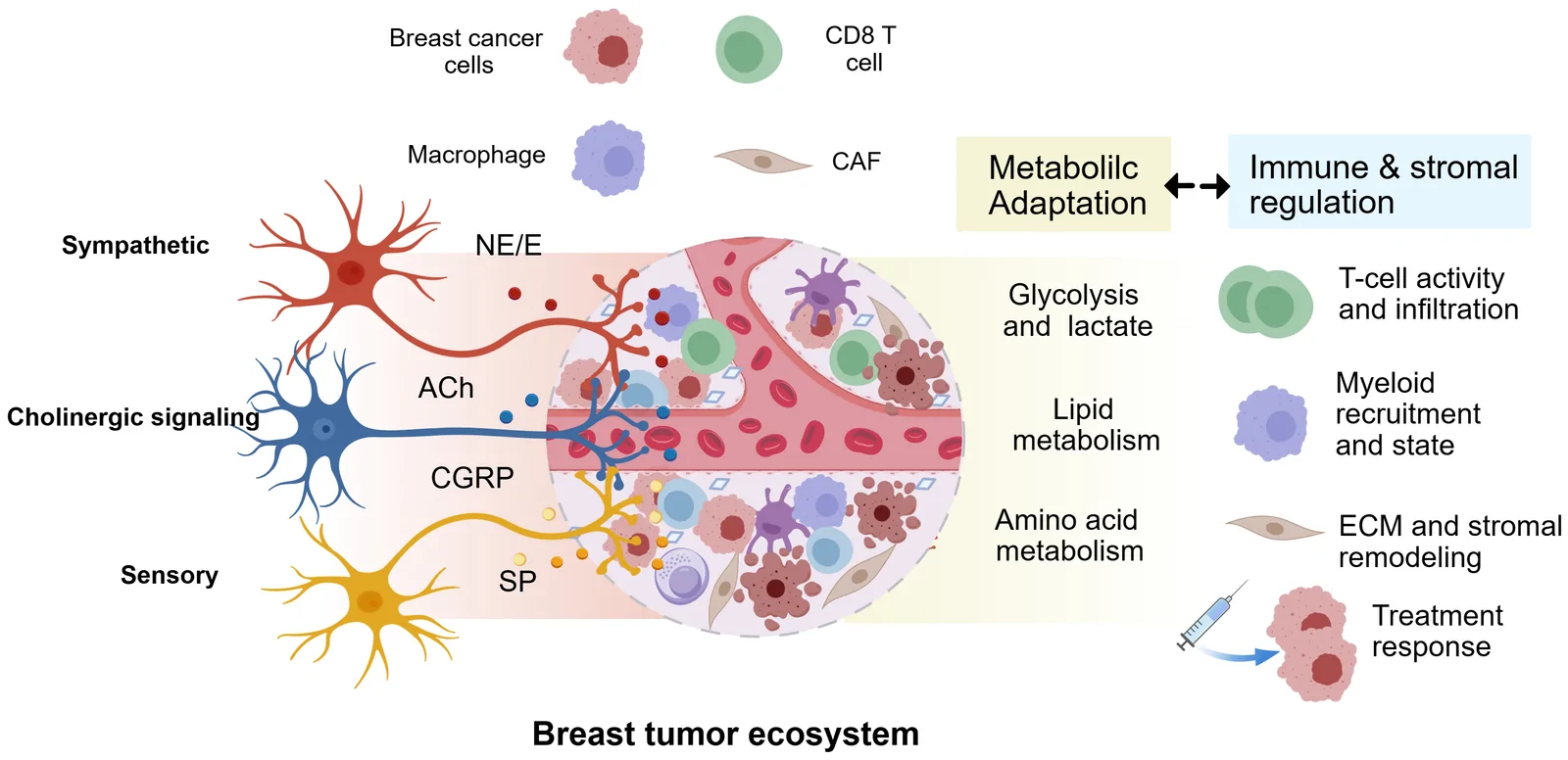
An overview of the neuro–metabolic–immune axis in breast cancer. Peripheral neural signaling can influence breast cancer cells, immune populations, and stromal cells through mediator-, receptor-, and cell-specific mechanisms. Sympathetic catecholamines, cholinergic signaling, and sensory neuropeptides intersect with tumor-cell programs and metabolic adaptations involving glucose and lactate metabolism, lipid metabolism, and amino acid utilization. Metabolic changes can further modify immune-cell activity and stromal organization, while tumor-, stromal-, and immune-derived signals may reciprocally influence neural remodeling. These interactions are context dependent and may vary according to receptor expression, responding cell type, breast cancer subtype, metabolic state, and treatment context. The neuro-metabolic-immune axis is therefore depicted as a network of interconnected interfaces rather than a single linear causal pathway. Created with BioGDP.com (133).

### 1.1 Literature identification and evidence assessment

To support this evidence-graded narrative synthesis, we conducted targeted searches of PubMed and Web of Science, with the final literature search performed on 1 October 2026. Search terms covered four domains: neural and neuroactive regulation in breast cancer; metabolic reprogramming; immune and stromal regulation; and therapeutic or clinical investigation. Representative terms included tumor innervation, adrenergic and cholinergic signaling, sensory neurons, Schwann cells, glycolysis, lipid metabolism, lactate and lactylation, glutamine metabolism, CD8 T cells, macrophages, CAFs, immune exclusion, β-blockers, neuropeptide antagonists, metabolic inhibitors, and clinical trials. ClinicalTrials.gov was additionally searched for relevant clinical studies. Primary studies conducted in breast cancer were prioritized, whereas cross-tumor and non-tumor findings were retained as complementary evidence. Clinical studies were interpreted according to study design, endpoint, target engagement or pharmacodynamic activity, and whether anticancer efficacy was directly evaluated.

To distinguish integrated causal evidence from contextual or extrapolated observations, we classified the literature into four evidence levels. Level I denotes an integrated neuro–metabolic–immune mechanism in which neural or neuroactive signaling, metabolic reprogramming, and an immune consequence were functionally linked within the same breast cancer model. Level II denotes experimentally demonstrated direct neural interfaces in breast cancer, including neural–metabolic and neural–immune interactions. Level III denotes breast cancer-specific metabolic or immune mechanisms for which the neural connection remains indirect or has not been experimentally established. Level IV comprises complementary evidence from other tumor types or non-tumor systems, as well as computational, transcriptomic, imaging, or associative observations without direct causal validation. Where relevant, whether the neuronal source or neural input was directly demonstrated is reported separately.

## 2 Functional regulation of the peripheral nervous system in breast cancer

Peripheral nerves are increasingly recognized as active components of the breast tumor microenvironment (TME). Sympathetic and sensory nerve fibers release neurotransmitters and neuropeptides that act on cancer cells, stromal populations, and immune cells, while cholinergic signaling may involve neuronal or non-neuronal sources of acetylcholine (9). Evidence is most extensive for sympathetic and sensory pathways, which are supported by human breast cancer observations and mechanistic preclinical studies, whereas evidence for cholinergic mechanisms derives mainly from receptor-level studies, often without an established neuronal acetylcholine source. Across these neural compartments, biological effects are determined by receptor subtype, responding cell population, and tumor context.

### 2.1 Autonomic nervous system signaling in breast cancer

Neural and cholinergic regulation within the breast TME includes catecholaminergic sympathetic signaling and acetylcholine-dependent receptor pathways. Sympathetic adrenergic signaling currently has the more extensive breast cancer-specific mechanistic support, whereas direct evidence for parasympathetic regulation remains limited.

#### 2.1.1 Sympathetic nervous system

Human pathological studies first established the clinical relevance of tumor innervation in breast cancer. PGP9.5-positive nerve fibers were detected in 61.8% of invasive ductal carcinomas and were associated with higher histological grade, shorter disease-free survival, and increased microvessel density (10). Notably, because PGP9.5 is a pan-neuronal marker, these findings demonstrate tumor innervation but do not specifically establish sympathetic innervation. More direct evidence was subsequently provided by an independent breast cancer cohort in which peripheral nerves were detected in approximately 85% of tumors, with the majority of nerve trunks and fibers showing positivity for the sympathetic marker tyrosine hydroxylase (TH). Higher nerve density was also associated with poorer progression-free and overall survival (11). These human tissue studies link sympathetic-rich tumor innervation with aggressive clinicopathological features and poorer clinical outcomes.

Preclinical studies provide stronger mechanistic evidence for adrenergic regulation of breast cancer progression. Chronic stress enhanced distant metastasis through β-adrenergic signaling and macrophage-dependent remodeling of the primary tumor microenvironment (20). Tumor-cell β2-adrenergic receptor (β2-AR) signaling also contributed directly to this phenotype: β2-AR knockdown reduced stress-enhanced metastasis in MDA-MB-231(HM) cells (21), whereas norepinephrine (NE) and epinephrine promoted migration and invasion through β2-AR-dependent Src activation (22). In the 4T1 model, chronic stress and NE further promoted VEGF/FGF2-dependent angiogenesis through β2-AR-mediated suppression of PPARγ (23). These studies provide breast cancer-specific mechanistic evidence linking β2-AR signaling to tumor-cell invasiveness, angiogenesis, and metastatic progression.

Adrenergic regulation also extends to the immune compartment through distinct cell-specific mechanisms rather than a single uniform immunosuppressive pathway. In MDA-MB-231 cells, NE-induced β2-AR/NF-κB signaling increased IL-6 secretion, which subsequently activated STAT3 and promoted M2-like polarization in macrophages, rather than NE acting directly on macrophages (24). Chronic stress also increased myeloid-derived suppressor cell accumulation through β-adrenergic activation of the IL-6/JAK/STAT3 pathway in a murine breast carcinoma model, an effect attenuated by propranolol or pathway blockade (25). More recently, genetic deletion of β2-AR, including ILC2-specific deletion, reduced the protumor activity of type 2 innate lymphoid cells, suppressed breast tumor growth, and was accompanied by enhanced T-cell responses (26). These findings indicate that sympathetic signaling can reshape antitumor immunity through distinct β2-AR-dependent pathways involving tumor cells, myeloid populations, and innate lymphoid cells. Recent evidence further suggests that adrenergic signaling in TNBC may participate in bidirectional tumor–nerve communication. Luo et al. reported that sympathetic adrenergic cells promoted TNBC-cell proliferation and reduced chemotherapy sensitivity through NE signaling, whereas TNBC cells increased TH expression and NE production in neural cells partly through tumor-derived NGF (27). Genetic or pharmacological inhibition of TH attenuated these effects, suggesting a potential positive-feedback circuit between TNBC cells and adrenergic neural signaling. However, its relevance in human disease awaits further *in vivo* and independent clinical validation.

Clinical evidence for β-adrenergic blockade remains less definitive. A population-based cohort and meta-analysis found no improvement in breast cancer-specific survival among patients with breast cancer overall, although more favorable survival associations were observed in the TNBC subgroup (28). These observational findings support further investigation but do not establish therapeutic efficacy. More broadly, most mechanistic evidence still derives from cell-based experiments and murine stress models, in which local sympathetic activity is difficult to distinguish from systemic catecholamine responses. This distinction will be important when translating adrenergic biology into breast cancer therapy.

Within the evidence hierarchy used in this review, experimentally demonstrated adrenergic–tumor and adrenergic–immune interactions in breast cancer are classified as Level II evidence, whereas population-level associations with β-blocker exposure are considered separately as clinical associative evidence.

#### 2.1.2 Cholinergic signaling

Compared with sympathetic adrenergic signaling, direct evidence for parasympathetic regulation of breast cancer remains limited. Acetylcholine (ACh), the neurotransmitter classically associated with parasympathetic signaling, acts through nicotinic (nAChRs) and muscarinic acetylcholine receptors (mAChRs). Because acetylcholine within the tumor microenvironment can also be produced by cancer and other non-neuronal cells, cholinergic receptor activity does not by itself demonstrate parasympathetic innervation (29, 30). Its biological consequences instead depend on receptor subtype, responding cell population, and breast cancer context.

One of the best-characterized immunoregulatory components of cholinergic signaling is the α7 nicotinic acetylcholine receptor (α7nAChR/CHRNA7). Earlier non-tumor studies established α7nAChR as a regulator of myeloid inflammatory responses (31). More recently, breast cancer-specific evidence has shown that CHRNA7 loss increases tumor burden, whereas pharmacological α7nAChR activation suppresses tumor progression in E0771, PyMT, and 4T1 models (32). This effect was associated with enhanced antigen-presenting cell activity, required CD8^+^ T cells, and was further strengthened by anti-PD-L1 therapy. Notably, these experiments manipulated the receptor rather than vagal activity itself; they therefore establish an α7nAChR-dependent immune mechanism but not a direct antitumor effect of parasympathetic innervation.

By contrast, cholinergic signaling in breast cancer cells can produce distinct and, in some settings, tumor-promoting effects. In ER-positive breast cancer cells, ACh activated MAPK/ERK and PI3K/Akt signaling and increased cell viability, whereas these responses were less evident in ER-negative MDA-MB-231 cells (33). Activation of α7nAChR also promoted proliferation and motility in MCF-7 cells (34), while nicotine reduced PD-L1/PD-L2 expression in HER2-positive SK-BR-3 cells but not in the TNBC models HCC1937 or MDA-MB-231 (35). Receptor specificity is further illustrated by α9nAChR/CHRNA9 in TNBC. α9nAChR has been implicated in nicotine-induced angiogenesis (36), and more recent evidence links elevated CHRNA9 to poorer outcome and demonstrates that CHRNA9 can promote TNBC-cell growth through proliferative and ferroptosis-regulatory pathways (37). Together, these observations show that the consequences of cholinergic signaling cannot be inferred from ACh exposure alone but instead depend on the receptor and cellular compartment engaged.

Non-neuronal ACh further complicates the interpretation of cholinergic activity as a surrogate for parasympathetic signaling. In a breast cancer metastasis model, chronic stress induced pulmonary epithelial cells to increase ACh production, which promoted neutrophil extracellular trap formation and remodeling of the lung pre-metastatic niche (30). Increased choline acetyltransferase expression was also detected in lung tissues from patients with breast cancer lung metastases. This mechanism therefore represents epithelial-derived cholinergic signaling rather than direct vagal neurotransmission.

Consistent with this distinction, direct evidence linking vagal activity to breast cancer control remains comparatively weak. Cervical vagotomy increased metastatic burden in a 4THMpc breast carcinoma model (38), but the mixed sensory and autonomic composition of the vagus nerve and accompanying changes in substance P preclude attribution of this effect specifically to parasympathetic cholinergic signaling. Clinically, higher heart-rate variability has been associated with longer survival in patients with metastatic or recurrent breast cancer (39), and similar associations have been reported across heterogeneous cancer cohorts (40); however, these observational measures do not establish that increasing vagal activity suppresses breast cancer. Current evidence therefore supports receptor- and cell-specific cholinergic mechanisms more strongly than a uniform antitumor role for parasympathetic innervation. Accordingly, receptor-dependent cholinergic mechanisms demonstrated in breast cancer are classified here as Level III when a neuronal acetylcholine source has not been established; vagal or parasympathetic regulation is discussed separately and is not inferred from cholinergic receptor activity alone.

### 2.2 Sensory nerves

Sensory nerves can influence breast cancer through both direct nerve–tumor interactions and the local release of neuropeptides, particularly calcitonin gene-related peptide (CGRP) and substance P (SP). Human TNBC tissues contain abundant sensory fibers within tumor-associated innervation (41, 42).In experimental models, breast cancer cells adhere to sensory neurites and migrate along neuronal processes, while co-culture with dorsal root ganglion neurons enhances TNBC-cell migration and adhesion-related transcriptional programs (42). Loss of the semaphorin receptor PlexinB3 attenuates this nerve-associated migration. These data support both structural and paracrine contributions of sensory innervation to breast cancer progression.

A particularly well-defined breast cancer-specific mechanism involves the SP–TACR1/NK1R axis. Padmanaban et al. showed that highly metastatic mammary tumors increase sensory innervation and stimulate neuronal SP release (43). SP acts on TACR1-expressing cancer cells, inducing death in a small TACR1-high population and releasing extracellular single-stranded RNAs that activate TLR7 in neighboring tumor cells, thereby initiating a prometastatic transcriptional program. Pharmacological TACR1 blockade with aprepitant suppressed tumor growth and metastasis in multiple breast cancer models (43). Human transcriptomic analyses further suggest that TLR7-associated neural programs are enriched in TNBC, although their relationship with outcome is non-linear and varies among molecular subgroups (44). The SP–TACR1–ssRNA–TLR7 pathway therefore defines a breast cancer-specific neural–tumor circuit whose clinical relevance may vary across TNBC molecular subgroups.

CGRP illustrates the importance of distinguishing breast cancer-specific mechanisms from cross-tumor evidence. Direct CGRP–RAMP1-mediated CD8^+^ T-cell dysfunction has been demonstrated primarily in melanoma models (45). In TNBC, by contrast, tumor-derived NGF stimulates sensory neurons to release CGRP, which acts predominantly on RAMP1-expressing cancer-associated fibroblasts (41).RAMP1 signaling enhances collagen deposition and extracellular-matrix remodeling, thereby promoting immune exclusion; disruption of sensory input or RAMP1 signaling remodels the stroma and improves the response to anti-PD-1 therapy in preclinical models. Thus, stromal remodeling currently represents the best-supported CGRP mechanism in breast cancer.

Recent studies further extend sensory-neuropeptide signaling beyond immune regulation. Makabe et al. linked persistent breast cancer-associated pain with higher circulating CGRP and SP levels and showed that activated human sensory neurons promote EMT, migration, and reduced docetaxel sensitivity in TNBC cells (46). Sensory-neuron activation also accelerated tumor growth in orthotopic and patient-derived models, whereas combined CGRP- and SP-receptor blockade attenuated these effects. However, sensory activation is not uniformly tumor promoting. Pharmacological activation of TRPV1-positive fibers increased intratumoral CD8^+^ T-cell recruitment and IFN-γ responses and reduced metastatic burden in a murine breast cancer model (47). These apparently divergent findings indicate that the consequences of sensory signaling depend on neuronal state, neuropeptide output, responding cell type, and experimental context.

Breast cancer-specific evidence supports sensory-neuron regulation of tumor migration through PlexinB3, metastatic signaling through the SP–TACR1–ssRNA–TLR7 axis, and stromal remodeling through the NGF–CGRP–RAMP1 pathway (41–43). These experimentally demonstrated breast cancer neural–tumor and neural–stromal/immune interactions are classified here as Level II evidence. By contrast, direct CGRP–RAMP1-mediated CD8^+^ T-cell dysfunction remains supported predominantly by non-breast tumor models and is therefore treated as Level IV complementary evidence (45). Overall, sensory nerves emerge as important but highly context-dependent regulators of tumor–stromal–immune interactions in breast cancer, particularly in TNBC.

### 2.3 Schwann cells in tumor-nerve and immune crosstalk

Schwann cells (SCs), the principal glial cells of the peripheral nervous system, can actively participate in tumor–nerve communication. In a mouse model of breast cancer-associated pain, tumor-derived Ref-1 altered Schwann-cell signaling and increased CXCL2 production, thereby promoting CXCR2-dependent macrophage recruitment into the sciatic nerve (48). Genetic depletion of Schwann-cell CXCL2 or pharmacological blockade of CXCL2-CXCR2 signaling reduced macrophage infiltration and nociceptive behavior. This study establishes a breast cancer-specific tumor-Schwann cell-macrophage interaction in the context of cancer pain.

Schwann cells have also been implicated in perineural invasion (PNI), although the relevant mechanistic evidence derives largely from non-breast cancer models. In experimental PNI systems, Schwann cell-derived CCL2 recruits CCR2^+^ inflammatory monocytes, which differentiate into macrophages and facilitate nerve invasion through cathepsin B-dependent disruption of the perineurial barrier (49). These studies define a Schwann cell–myeloid mechanism of neural invasion in non-breast cancer models; whether the same pathway operates in breast cancer has not yet been directly tested.

More recently, human TNBC data have linked Schwann cell-associated features to a distinct stromal and immune context. A Schwann cell-related transcriptomic score, spatially localized to intratumoral nerve regions, was associated across multiple TNBC cohorts with reduced cytolytic activity and immune-cell infiltration together with enrichment of TGF-β, epithelial-mesenchymal transition, and stromal-remodeling programs (50). Associations with pathological complete response and survival varied across cohorts. These data position Schwann cells as a candidate interface between peripheral nerves, tumor-associated inflammation, and stromal remodeling in TNBC (**Figure 2**). Hence, the tumor–Schwann cell–CXCL2–macrophage interaction demonstrated in a breast cancer pain model is considered Level II evidence within that specific biological context, whereas the CCL2-dependent perineural-invasion mechanism and Schwann cell-associated transcriptomic signatures are classified as Level IV complementary or associative evidence.

**Figure 2:**
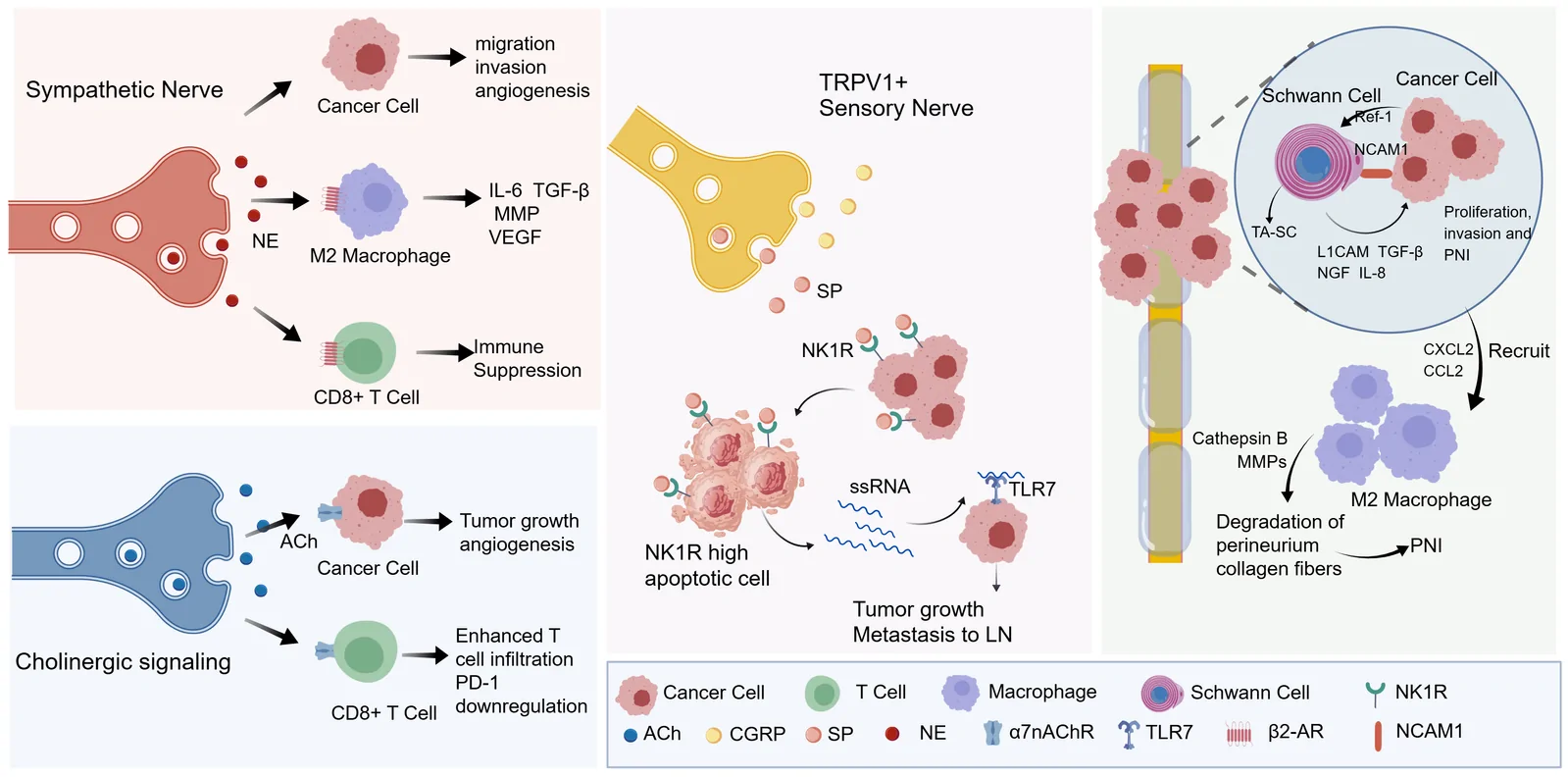
Peripheral neural systems and cell-specific signaling in the breast cancer microenvironment. Sympathetic, cholinergic, sensory-neuropeptide, and Schwann-cell-associated pathways can influence breast cancer cells and components of the tumor microenvironment through cell- and receptor-dependent mechanisms. Sympathetic norepinephrine (NE) signaling is associated with tumor-cell migration, invasion, and angiogenesis and can also modulate macrophage and CD8^+^ T-cell states. Acetylcholine (ACh)-dependent cholinergic signaling exerts context-dependent effects on both breast cancer cells and immune populations. Sensory nerves release neuropeptides, including substance P (SP) and calcitonin gene-related peptide (CGRP); the SP–NK1R/TACR1 pathway can promote a prometastatic program through the release of extracellular single-stranded RNA and activation of TLR7 in neighboring tumor cells. Schwann cells undergo tumor-associated reprogramming and participate in reciprocal signaling with cancer cells, while Schwann-cell-derived chemokines can recruit macrophages and contribute to extracellular-matrix remodeling and perineural invasion. The biological consequences of these pathways depend on receptor subtype, responding cell type, and disease context, and some Schwann-cell and immune mechanisms shown are supported primarily by complementary or cross-tumor evidence. Created with BioGDP.com (133).

### 2.4 Additional neuroactive and neural guidance signals in breast cancer

Beyond adrenergic, cholinergic, and sensory-neuropeptide signaling, several additional neurotransmitters, neurotrophic factors, and axon-guidance molecules have been implicated in breast cancer biology. These include dopamine, serotonin, γ-aminobutyric acid (GABA), neurotrophins, netrins, semaphorins, Eph/ephrin molecules, and Slit proteins. Importantly, many of these signals can originate from cancer, stromal, or immune cells rather than nerve terminals; their presence within the tumor microenvironment therefore does not necessarily imply direct neuronal regulation. Their relevance to a neuro–metabolic–immune framework should consequently be interpreted according to cellular source and evidence context.

#### 2.4.1 Additional neurotransmitter systems

Dopamine provides breast cancer-specific evidence for neuroactive receptor signaling, although not yet for functional dopaminergic innervation. In an immunocompetent 4T1 model, dopamine acted through dopamine receptor D1 to suppress cancer stem-like features and PD-L1 expression, enhance cytotoxic T-cell activity, and improve the response to CTLA-4 blockade (51). More recently, stimulation of ProkR2-expressing sensory neurons in the sciatic nerve suppressed 4T1 tumor growth by enhancing intratumoral NK-cell cytotoxicity through D1-like dopamine receptor–cAMP–PKA–CREB signaling and further improved the response to PD-1 blockade (52). This study provides Level II evidence that experimentally induced peripheral neural activity can engage dopaminergic signaling to regulate antitumor immunity in TNBC, but it does not establish the presence of functional dopaminergic nerve terminals within the breast tumor itself.

Serotonin and GABA have also been implicated in breast cancer progression and tumor–immune regulation. Serotonin-related pathways have been implicated in breast cancer stem-cell biology (53).More recently, Paixão et al. showed that 5-HT enhanced proliferation and migration in MCF-7 cells and altered ER, PR, and HER2 expression, while fluoxetine exposure accelerated tumor growth in a murine model (54). These findings strengthen breast cancer-specific evidence for serotonergic regulation but do not establish direct serotonergic innervation. Evidence that SERT constrains CD8^+^ T-cell activity and limits responses to PD-1 blockade remains derived primarily from broader solid-tumor models (55).

GABA-related evidence in breast cancer has also expanded beyond transcriptomic association. In addition to single-cell evidence identifying GABA-associated programs in metastatic breast cancer (56), Wang et al. reported that GAD1-driven GABA production in MDA-MB-231 cells enhanced tumor-cell proliferation and invasion, promoted M2-like macrophage features, impaired CD8^+^ T-cell effector readouts, and enhanced angiogenic responses (57). Because these experiments implicate tumor-cell-derived rather than neuron-derived GABA, they support a breast-cancer-specific GABA-tumor/immune mechanism without establishing direct neural input. Consequently, these breast cancer serotonin- and GABA-associated mechanisms are classified as Level III evidence, whereas cross-tumor immune mechanisms are retained as Level IV complementary evidence.

#### 2.4.2 Neurotrophic and axon-guidance signals

Neurotrophic factors can mediate reciprocal communication among tumor cells, immune populations, and peripheral nerves. The NGF–sensory neuron–CGRP pathway in TNBC has been discussed above (41). More recently, tumor-associated macrophages were identified as a major source of BDNF in experimental TNBC (58). Loss of immune-derived BDNF reduced sensory tumor innervation and tumor growth, whereas transfer of BDNF-competent macrophages restored both phenotypes. Conversely, sensory-nerve depletion also suppressed tumor growth. These findings define a macrophage–BDNF–sensory nerve circuit and demonstrate that neuro–immune communication in breast cancer can proceed bidirectionally rather than exclusively from nerves to immune cells.

Axon-guidance molecules can likewise be co-opted by breast tumors independently of their classical neuronal functions. Netrin-1 blockade reduced cancer stem-like properties, delayed post-chemotherapy relapse, and enhanced CTLA-4 blockade in breast cancer models, accompanied by reduced polymorphonuclear MDSC recruitment (59). SEMA4C has been linked to macrophage recruitment and angiogenesis, whereas SEMA7A promotes endocrine resistance and metastatic progression (60, 61). These findings demonstrate tumor- and immune-regulatory functions of neural-guidance molecules independently of a demonstrated neuronal source.

Among these pathways, Slit2 provides a particularly relevant link to immune-cell metabolism. In breast cancer models, Slit2 shifted macrophage metabolism toward increased glycolysis and reduced fatty-acid oxidation, promoted an M1-like phenotype and phagocytic activity, and suppressed tumor growth and metastasis (62, 63). Higher Slit2 expression in patient samples was also associated with more favorable survival and lower CD163^+^ macrophage abundance (63). These findings support a role for Slit2 in macrophage metabolic and functional reprogramming within the breast tumor microenvironment, independent of a demonstrated neural source.

At the clinical level, a neuron-projection-guidance (NPG) transcriptional signature evaluated across 9,017 patients was associated with reduced immune-effector activity, lower pathological complete response rates, and poorer outcomes, particularly in TNBC (64). Because the NPG score is a transcriptional surrogate, it should be interpreted as a marker of neural-guidance-associated tumor biology rather than a direct measure of innervation. Across these studies, neuroactive and neural-guidance pathways broaden the mechanistic landscape of breast cancer, with their relevance determined by cellular source and disease context (**Table 1**).

**Table 1:** Key neuroactive and neural-guidance signals in breast cancer: receptors, biological effects, and evidence context.

Neuroactive molecules	Major receptor(s)	Key biological effects	Evidence context/model	Direct neural involvement	Evidence level	Reference
Norepinephrine (NE)/Epinephrine (E)	β2-AR on tumor cells, myeloid cells and ILC2s; β1-AR on T-cells	Promotes tumor invasion and angiogenesis; modulates myeloid, ILC2, and T-cell responses, including β2-AR-dependent metabolic reprogramming of MDSCs	Human BC observational; BC preclinical; β1-AR-associated T-cell exhaustion:mainly cross-tumor/chronic-disease models	Partial/Mixed	I for β2-AR–MDSC immunometabolism; II for other BC adrenergic interfaces; IV for β1-AR exhaustion	(20, 21, 23–26, 73, 93)
Acetylcholine	α7 nAChR and α9 nAChR on immune and tumor cells; mAChRs on tumor cells	α7 nAChR signaling in APC can enhance CD8^+^ T-cell antitumor immunity, whereas cholinergic receptor signaling in tumor cells regulates proliferation, migration, angiogenesis, and ferroptosis sensitivity	BC preclinical/*in vitro*; human autonomic observational evidence;	No/indirect	III; IV for autonomic associative evidence	(30, 32–37, 39)
Dopamine	D1R on NK cells;DRD1 on TNBC stem-like cells	Suppresses CSC properties and PD-L1 expression, with increased CTL infiltration and activity.	BC preclinical	Mixed	II for nerve stimulation–NK pathway; III for pharmacological DRD1 effects	(51, 52)
Serotonin	5-HTRs on breast cells; SERT on T cells(cross-tumor models)	5-HT7 promotes TNBC proliferation and invasion; 5-HT5A supports breast tumor-initiating cell maintenance; SERT limits CD8^+^ T-cell autocrine serotonin signaling in other tumor models	BC preclinical; cross-tumor evidence	No	III for BC tumor-cell signaling; IV for SERT–T-cell mechanism	(55, 127, 128)
GABA	GABA_B_ receptor on cancer cells(cross-tumor models)	BC: GAD1/GABA-associated tumor growth, immunosuppression, and angiogenesis. Cross-tumor: GABA_B–β-catenin signaling suppresses CCL4/CCL5 and immune-cell infiltration.	BC *in vitro*/co-culture experiments; BC computational analysis; cross-tumor preclinical models	No	III;IV	(56, 57, 129)
Substance P (SP)	TACR1/NK1R on breast cancer cells	Activates the SP–TACR1–ssRNA–TLR7 prometastatic pathway and may influence tumor-cell plasticity and treatment response	BC preclinical: mechanistic; human TNBC: associative/transcriptomic support	Yes	II	(43, 44, 46)
CGRP	CALCRL/CLR–RAMP1 on CAFs in TNBC; RAMP1 on CD8^+^ T cells (cross-tumor models)	Promotes CAF-mediated collagen deposition and immune exclusion in TNBC; direct CD8^+^ T-cell suppression(cross-tumor models)	BC preclinical; cross-tumor evidence	Yes	II for TNBC CGRP–CAF; IV for direct CD8 effects	(41, 45, 46)
NGF	TrkA/NTRK1 on sensory neurons; p75NTR/NGFR and TrkA on breast cancer cells	Promotes sensory-neuron activation and CGRP release in TNBC; NGF signaling can also support breast cancer-cell survival	BC preclinical; human BC supportive evidence	Yes	II for sensory pathway; III for tumor-cell-only effects	(41, 122)
BDNF	TrkB/NTRK2 on sensory neurons	Macrophage-derived BDNF promotes sensory axonogenesis and supports breast tumor progression	BC preclinical	Yes	II	(58)
Netrin-1	UNC5B on breast cancer cells	Supports CSC/TIC properties and treatment resistance; netrin-1 blockade reduces PMN-MDSC recruitment and improves responses to chemotherapy and immune checkpoint blockade	Human BC associative; BC preclinical	No	III	(59)
SEMA4C	Plexin-B2 on breast cancer cells	Promotes tumor-cell self-renewal and induces angiogenin and CSF-1 production, thereby enhancing macrophage recruitment and angiogenesis	BC preclinical;human BC supportive evidence	No	III	(60)
SEMA7A	Integrins β1/β4 on ER^+^ breast cancer cells	Activates prosurvival signaling and promotes endocrine-therapy resistance and metastatic progression	Human ER^+^ BC associative; BC preclinical	No	III	(61, 130)
Eph receptor signaling (EPHA2)	EPHA2 on TNBC tumor cells	Promotes proliferation and invasion and suppresses pyroptosis through PI3K/AKT/mTOR-associated signaling	TNBC preclinical and BC computational evidence	No	III	(131)
Slits	Robo Receptors on macrophages and breast cancer cells	Promotes M1-like phagocytic and antifibrotic macrophage activity, suppresses IL-6 and fibrosis, and reduces breast tumor growth and metastasis; macrophage metabolic reprogramming	BC preclinical; human BC associative/supportive evidence	No	III	(62, 63)

Within this hierarchy, the macrophage–BDNF–sensory nerve circuit represents Level II breast cancer evidence, whereas neuroactive or neural-guidance pathways whose neuronal source has not been demonstrated, including Netrin-, Semaphorin-, Eph-, and Slit-associated mechanisms, are classified predominantly as Level III. Transcriptomic neural-guidance signatures are considered Level IV associative evidence.

## 3 Neural interfaces with tumor metabolism in breast cancer

Metabolic reprogramming is a well-established feature of breast cancer, but the extent to which these metabolic changes are directly controlled by neural signals varies considerably among metabolic pathways. Adrenergic signaling provides the clearest breast cancer-specific evidence linking neural or neuroendocrine activity to glucose and lipid metabolism, whereas brain-metastatic models have revealed additional direct neural–metabolic interfaces involving glutamate, GABA, and neuron–astrocyte metabolic coupling. More recently, direct mitochondrial transfer from cancer-associated neurons has identified organelle exchange as another mechanism through which neural inputs can alter breast cancer bioenergetics. Moreover, many metabolic alterations discussed in the context of neural signaling can also arise independently through oncogenic, stromal, or immune-cell programs. In this section, we therefore focus on metabolic pathways for which a plausible neural interface has been identified and distinguish direct breast cancer evidence from broader metabolic or cross-tumor observations (**Figure 3**).

**Figure 3:**
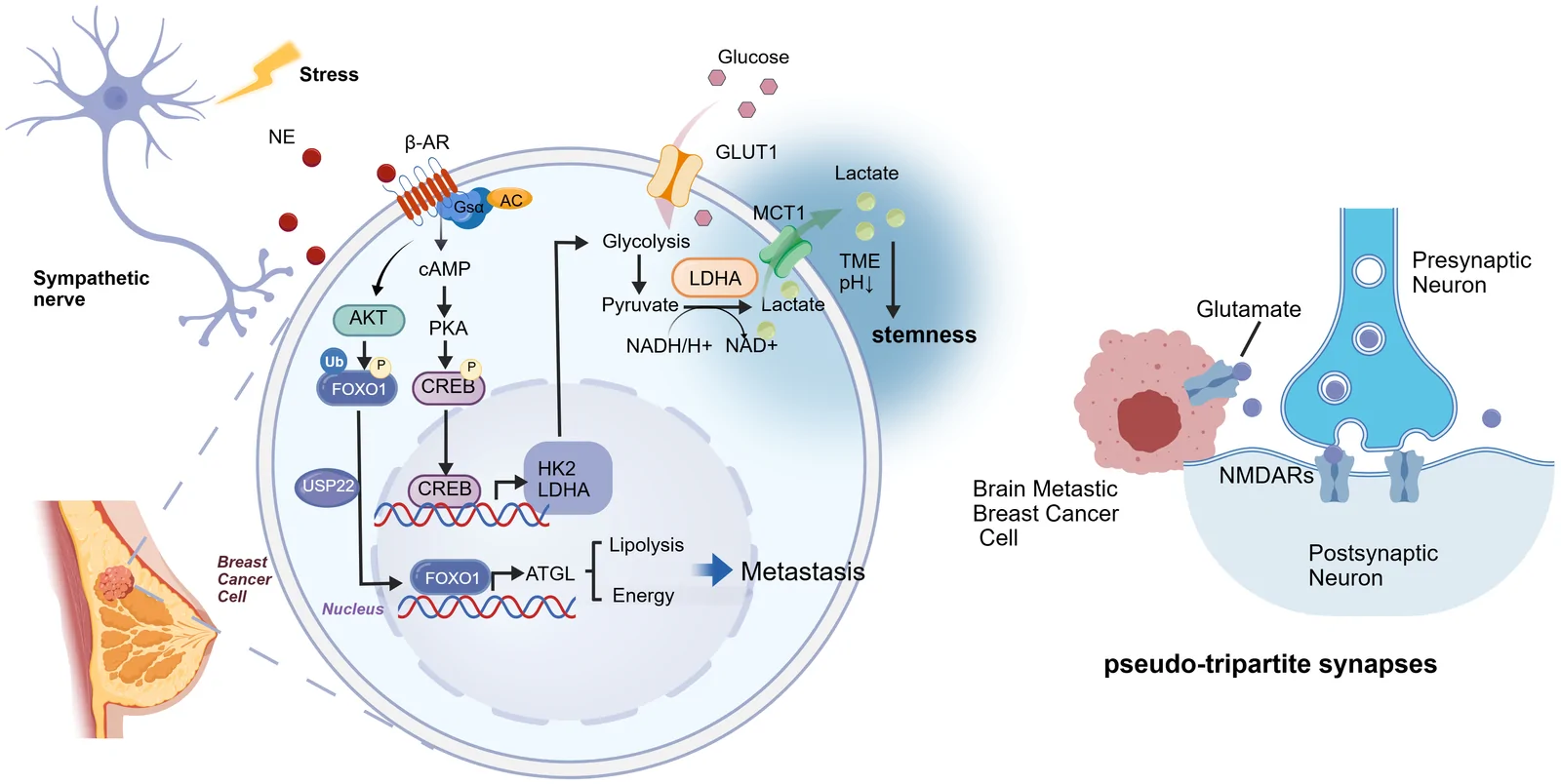
Neural interfaces with glucose, lipid, and amino acid metabolism in breast cancer. Stress-associated adrenergic signaling can modify glucose metabolism in breast cancer through experimentally supported effects on HK2 and LDHA. β-Adrenergic regulation of HK2 influences glucose utilization and tumor 18F-FDG uptake, whereas chronic stress and epinephrine increase LDHA-dependent glycolysis and lactate production, which can promote USP28–MYC–SLUG-associated stem-like programs. Adrenergic signaling can also promote lipolysis in breast cancer cells through the AKT–USP22–FOXO1–ATGL pathway, supporting metastatic progression. In the brain metastatic niche, breast cancer cells exploit neuronal metabolites and reciprocally remodel neural metabolism. Neuron-derived glutamate activates NMDAR/GluN2B signaling in metastatic breast cancer cells, whereas tumor-derived extracellular-vesicle miR-199b-5p suppresses metabolic transporters in astrocytes and neurons, increasing extracellular retention of glutamate, glutamine, and lactate. Created with BioGDP.com (133).

### 3.1 Adrenergic and neuroactive regulation of glucose metabolism

Aerobic glycolysis is a prominent metabolic feature of breast cancer, but direct evidence linking neural signaling to this phenotype is concentrated primarily on adrenergic pathways. An early breast cancer study showed that β-adrenergic receptor signaling can influence glucose utilization through regulation of hexokinase 2 (HK2) (15). In 4T1, MDA-MB-231, and MCF-7 breast cancer cells, β-adrenergic receptors were detectable, and pharmacological β-adrenergic blockade with propranolol reduced HK2 protein expression. In 4T1 tumor-bearing mice, propranolol also reduced tumor ^18^F-FDG uptake and HK2 expression, whereas GLUT1 expression was not consistently altered (15). These findings provide breast cancer-specific evidence that β-adrenergic signaling can modulate glycolytic activity, although they do not support uniform regulation of all major glycolytic enzymes.

A second and more extensively characterized pathway involves stress-induced epinephrine and LDHA. Cui et al. demonstrated that chronic stress and epinephrine increased glucose uptake, extracellular acidification, glycolytic metabolites, and lactate production in breast cancer models (16). Epinephrine increased LDHA expression and promoted an LDHA-dependent metabolic shift toward glycolysis in both MCF-7 and MDA-MB-231 cells. Increased lactate production subsequently facilitated USP28-dependent stabilization of MYC and activation of the stemness-associated transcription factor SLUG, thereby promoting breast cancer stem-like properties (16). Silencing LDHA attenuated epinephrine-induced USP28/MYC signaling, supporting a functional role for LDHA in this stress-associated metabolic response. Notably, this pathway was demonstrated using breast cancer cell and xenograft models and therefore provides direct preclinical evidence linking stress-associated catecholamine signaling to glycolytic remodeling in breast cancer. Accordingly, the β-adrenergic–HK2 and epinephrine–LDHA pathways are classified as Level II evidence.

Neuroactive regulation of breast cancer metabolism is not restricted to adrenergic signaling. Serotonin has also been shown to increase glycolytic activity in breast cancer cells through 5-HT receptor-dependent JAK1/STAT3 and ERK1/2 signaling associated with PKM2 regulation, while a parallel adenylate cyclase/PKA pathway promotes mitochondrial biogenesis (65). Because a neuronal serotonin source was not established, this mechanism is classified as Level III evidence.

Complementary evidence from other malignancies suggests that β2-adrenergic signaling can extend to HIF-1α-dependent glycolytic programs, while increased glycolytic activity may further shape antitumor immunity through nutrient competition and the accumulation of metabolic by-products (66–68). These observations are retained as Level IV contextual evidence.

Thus, breast cancer-specific evidence supports adrenergic regulation of selected glycolytic nodes, particularly HK2 and LDHA, whereas other neuroactive–metabolic mechanisms remain less directly linked to neural input.

### 3.2 Adrenergic regulation of lipid metabolism in the breast tumor microenvironment

Lipid metabolism represents another metabolic interface through which neural and stress-associated signals may influence breast cancer progression. The breast tumor microenvironment is highly enriched in adipocytes, which can undergo metabolic remodeling and provide fatty acids to neighboring cancer cells (69). Among neural-associated signals, adrenergic activation provides the strongest evidence linking stress responses to lipid mobilization in breast cancer. Stress-associated epinephrine signaling can promote lipolysis within breast cancer cells, thereby supporting metastatic progression (17).

A direct mechanistic link between adrenergic signaling and lipid metabolism in breast cancer was demonstrated by Zhou et al., who identified an AKT–USP22–FOXO1–ATGL lipolytic circuit in breast cancer cells (17). Mechanistically, epinephrine induced AKT-dependent phosphorylation and stabilization of USP22, which deubiquitinated and stabilized FOXO1, thereby increasing ATGL expression and promoting tumor-cell lipolysis. Genetic or pharmacological inhibition of USP22 reduced epinephrine-induced lipolysis and metastatic progression (17). These findings provide direct evidence that stress-associated catecholamine signaling can regulate lipid metabolism within breast cancer cells.

Beyond tumor-cell lipid metabolism, lipid metabolic reprogramming also shapes immune-cell function within the tumor microenvironment. Increased fatty-acid oxidation (FAO) has been associated with suppressive immune populations, including MDSCs and regulatory T cells, across several tumor settings (70–72). Importantly, breast cancer-specific evidence extends this principle to adrenergic regulation of MDSC metabolism. In the 4T1 mammary tumor model, β2-AR signaling in MDSCs shifted metabolism from glycolysis toward oxidative phosphorylation and FAO, increased CPT1A expression, and enhanced autophagy and arachidonic-acid/PGE2 metabolism (73). These metabolic changes supported MDSC-mediated immunosuppressive function, providing Level I evidence for an integrated adrenergic–metabolic–immune mechanism in breast cancer.

Neurotrophic regulation may represent an additional interface, as NGF–TrkA signaling contributes to adipose-tissue innervation and metabolic homeostasis, although its direct role in breast cancer-associated adipose remodeling remains unclear (74).

Overall, adrenergic regulation of lipid metabolism is supported across both tumor-cell and myeloid compartments in breast cancer, whereas neurotrophic regulation of adipose metabolism remains less directly established.

### 3.3 Neural–metabolic adaptation in the brain metastatic niche

A particularly well-defined neural–metabolic interaction has been identified in breast cancer brain metastasis, where metastatic cells can exploit neuron-derived glutamate signaling. Zeng et al. showed that breast-to-brain metastatic cells express N-methyl-D-aspartate receptors (NMDARs), particularly GluN2B-containing receptors, but produce insufficient glutamate to sustain receptor activation. Instead, these cells establish pseudo-tripartite synapse-like contacts with glutamatergic neurons and utilize neuron-derived glutamate to activate NMDAR signaling, thereby facilitating metastatic colonization of the brain (18). This mechanism provides Level II evidence for a direct neural–metabolic interface.

More recent biochemical and molecular-imaging studies provide spatial support for this relationship. Tang et al. identified regional associations among glutamate/glutamine abundance, phosphorylated GluN2B, and glutamine uptake in breast cancer brain metastases (75). These observations provide Level IV supportive evidence for spatial coupling between glutamatergic signaling and metabolic adaptation.

A complementary breast cancer mechanism involves increased utilization of extracellular glutamate without direct demonstration of its neuronal source. Di Russo et al. showed that brain-tropic breast cancer cells upregulate the glutamate transporters EAAT1 and EAAT2 and exploit glutamate as an energy substrate supporting both oxidative phosphorylation and glycolysis (76). Inhibition of glutamate uptake reduced migratory and invasive behavior and decreased brain metastatic burden *in vivo*. Because direct neuronal delivery of glutamate was not demonstrated, this mechanism is classified as Level III evidence.

Breast cancer cells can also reshape neural metabolism to increase local nutrient availability, indicating that neural–metabolic communication is bidirectional. Ruan et al. showed that extracellular vesicles released by brain-tropic breast cancer cells are enriched in miR-199b-5p, which targets the glutamate transporter SLC1A2/EAAT2 in astrocytes and the glutamine and lactate transporters SLC38A2/SNAT2 and SLC16A7/MCT2 in neurons (19). Suppression of these transporters disrupted physiological neuron–astrocyte metabolic coupling and reduced the uptake of glutamate, glutamine, and lactate by brain-resident cells, resulting in extracellular retention of these metabolites and enhanced growth of metastatic breast cancer cells (19). This reciprocal tumor-to-neural metabolic interaction provides Level II evidence.

GABA, as a classic neurotransmitter, can be utilized by breast cancer metastatic cells as a metabolic substrate. Neman et al. showed that breast cancer brain metastases acquire a GABAergic phenotype, with increased expression of GABA receptors, transporters, and GABA transaminase (77). These metastatic cells can take up extracellular GABA and metabolize it through the GABA shunt to generate succinate and NADH, thereby providing additional metabolic support for tumor-cell growth (77). Subsequent work by Deshpande et al. showed that exposure to neurons further enhances neurotransmitter-responsive programs in breast cancer cells and increases their dependence on GABA from the surrounding neural microenvironment during early brain colonization (78). Together, these studies establish a Level II neural–metabolic interface in which brain-metastatic breast cancer cells acquire dependence on extracellular, including neuron-derived, GABA as a metabolic resource. Its relevance outside the brain metastatic niche remains unclear.

Collectively, these studies show that neural–metabolic communication in breast cancer brain metastasis is not restricted to a single neurotransmitter pathway. Rather, metastatic cells can exploit neuron-derived metabolic resources, reprogram neuron–astrocyte metabolic coupling, and adapt to metabolic support from neural-resident cells, with the strength of direct neural involvement varying across mechanisms.

### 3.4 Neuronal mitochondrial transfer and tumor bioenergetic plasticity

A distinct neural–metabolic mechanism involves direct organelle transfer from cancer-associated neurons to tumor cells. Hoover et al. showed that neuronal interactions enhance cancer-cell metabolic plasticity and that cancer-associated neurons can transfer mitochondria directly to adjacent breast cancer cells (79). Using MitoTRACER-based lineage recording, the authors demonstrated nerve-to-cancer mitochondrial transfer in 4T1 breast cancer models and showed that recipient tumor cells acquire enhanced mitochondrial metabolic capacity and stress resistance and become selectively enriched during metastatic progression. Unlike neurotransmitter- or neuropeptide-mediated signaling, this mechanism involves the direct transfer of metabolic machinery across the nerve–tumor interface. This mechanism provides Level II evidence for a direct neural–metabolic interface with experimentally demonstrated neuronal input.

## 4 The neuro-metabolic-immune axis

Neural, metabolic, and immune regulation in breast cancer converge at several experimentally supported interfaces rather than through a single obligatory cascade. Among these, lactate-associated metabolic and epigenetic signaling, adrenergic regulation of T-cell metabolism and function, and sensory-neuropeptide-mediated stromal remodeling provide particularly informative examples. These pathways illustrate how neural activity can modify tumor, stromal, or immune states, while tumor-derived metabolic signals can in turn communicate with peripheral nerves. These interfaces differ in the extent to which neural, metabolic, and immune components have been linked within the same experimental system. Fully integrated mechanisms remain uncommon, whereas pairwise neural–metabolic or neural–immune interactions and metabolic–immune mechanisms with indirect neural links are more widely documented. Together, the available evidence supports a convergent network of context-dependent interactions rather than a single linear pathway.

### 4.1 Lactate as a metabolic and epigenetic signal

Lactate is increasingly recognized as more than a terminal product of glycolysis and can function as both a metabolic substrate and a signaling molecule within the tumor microenvironment. In highly glycolytic breast tumors, lactate is exported predominantly through monocarboxylate transporters, contributing to extracellular acidification and metabolic competition that can impair antitumor immune-cell function (80, 81). Lactate can also participate directly in tumor–nerve communication. In a breast cancer bone-metastasis model, Okui et al. showed that MCT4-dependent lactate release from bone-colonizing breast cancer cells activated GPR81 in sensory neurons, increased neuronal ERK/CREB signaling and calcium responses, and contributed to cancer-associated bone pain (82). This tumor-to-sensory-neuron pathway provides Level II evidence for a direct metabolic–neural interface in breast cancer.

As discussed in Section 3.1, stress-associated epinephrine can enhance LDHA-dependent glycolysis and lactate production in breast cancer. Separately, breast cancer-specific studies have consistently linked tumor-derived lactate to macrophage polarization and immune dysfunction. Lactate promotes ERK/STAT3-dependent M2-like polarization and tumor-promoting macrophage activity (83), while multi-omics and *in vitro* analyses further showed that glycolysis-derived lactate suppresses M1-associated macrophage programs (84). More recently, lactate-induced TGF-β/SNAIL signaling was linked to M2-like polarization and impaired CD8^+^ T-cell cytotoxicity, together with increased exhaustion-associated markers (85). These studies establish a breast cancer-specific metabolic–immune branch centered on lactate.

Lactate can additionally influence gene regulation through lysine lactylation. The initial description of histone lactylation showed that increased intracellular lactate is associated with histone lysine lactylation and induction of reparative genes such as Arg1 in macrophages (86). Because this macrophage-lactylation mechanism was established outside breast cancer, it is retained here as complementary evidence rather than as a breast cancer-specific causal mechanism.

Studies in Breast cancer have further established tumor-intrinsic roles for histone lactylation. H4K79la and H4K91la are enriched in breast cancer tissues and can promote the transcription of glycolysis-related genes, including LDHA, PGK1, and HK1, thereby reinforcing glycolytic activity (87). More recent studies have extended this concept to additional lactylation sites, including H3K18la, and have linked lactylation-associated chromatin changes to metabolic and immune-related transcriptional programs in breast cancer (88, 89).

A recent TNBC study further connected lactate-dependent epigenetic regulation to immune evasion. PGK1-driven glycolysis increased lactate accumulation and H3K18 lactylation at the CCL5 promoter, thereby promoting CCL5-dependent MDSC recruitment and impairing CD8^+^ T-cell function (90). Pharmacological PGK1 inhibition reduced MDSC infiltration and enhanced the response to anti-PD-1 therapy. This mechanism provides Level III evidence for a breast cancer-specific metabolic–immune interface.

Taken together, lactate represents a point of convergence between metabolic, neural, and immune regulation in breast cancer, but the available evidence derives from parallel metabolic–neural and metabolic–immune mechanisms rather than from a single experimentally demonstrated neuro–metabolic–immune cascade.

### 4.2 Adrenergic regulation of immune-cell metabolism and function

As discussed in Section 3.2, β2-AR-dependent metabolic reprogramming of MDSCs provides Level I evidence for an integrated adrenergic–metabolic–immune mechanism in breast cancer (73). Adrenergic signaling can also directly influence the metabolic requirements of activated CD8^+^ T cells. Qiao et al. showed that stimulation of murine CD8^+^ T cells with the β-adrenergic agonist isoproterenol reduced GLUT1 expression, glucose uptake, and glycolytic activity following T-cell activation (91). These effects were dependent on β2-adrenergic receptor (β2-AR/ADRB2) signaling, as they were absent in Adrb2-deficient T cells and were reversed by propranolol. β2-AR activation also impaired the activation-associated increase in mitochondrial mass and mitochondrial respiration (91). Because these metabolic experiments were not performed within a breast cancer-specific tumor system, they are retained as Level IV complementary evidence for adrenergic regulation of T-cell immunometabolism.

In the 4T1 mammary carcinoma model, physiological or pharmacological reduction of β-adrenergic signaling increased the proportion of intratumoral CD8^+^ T cells with an effector phenotype, decreased PD-1 expression, and enhanced the response to anti-PD-1 therapy (92). The antitumor effect of propranolol was lost following CD8^+^ T-cell depletion, supporting a functional contribution of CD8^+^ T cells to β-blocker-mediated tumor control (92). These findings provide Level II breast cancer-specific evidence for an adrenergic–immune interface.

A distinct β1-adrenergic pathway has also been implicated in CD8^+^ T-cell exhaustion. Globig et al. showed that ADRB1 is upregulated in exhausted CD8^+^ T cells and that catecholamine–ADRB1 signaling increases intracellular cAMP, impairs T-cell receptor signaling, and promotes terminal exhaustion (93). Genetic or pharmacological disruption of ADRB1 improved T-cell function and responses to immune checkpoint blockade. This mechanism has been defined primarily in chronic infection, melanoma, and pancreatic cancer models and is therefore retained as Level IV complementary evidence.

Together with β2-AR-dependent metabolic regulation of MDSCs, these complementary studies support cell-type-specific adrenergic control of immune-cell metabolism and function. β2-AR signaling can restrict activation-associated metabolic reprogramming, whereas β1-AR signaling has been linked to chronic T-cell exhaustion; breast cancer models further demonstrate that reducing β-adrenergic signaling enhances intratumoral CD8^+^ T-cell activity and responsiveness to checkpoint blockade. Although these mechanisms have not yet been demonstrated simultaneously within human breast tumors, they provide a coherent mechanistic basis for adrenergic regulation of antitumor immune fitness.

### 4.3 Sensory neuropeptides in tumor adaptation and immune remodeling

Sensory neuropeptides can reshape the breast tumor microenvironment through stromal and immune-dependent mechanisms. In TNBC, tumor-derived NGF stimulates sensory neurons to release CGRP, which acts predominantly on RAMP1-expressing cancer-associated fibroblasts (CAFs) (41). Activation of the RAMP1–cAMP–PKA–CREB1 pathway enhances collagen production and promotes the formation of a dense extracellular matrix, thereby limiting immune-cell accessibility. Targeting sensory neurons or RAMP1 signaling reduced stromal remodeling and enhanced the response to anti-PD-1 therapy in preclinical TNBC models (41).

Sensory neuropeptides can also influence tumor-cell plasticity and treatment response. Makabe et al. showed that conditioned medium from activated human sensory neurons promoted epithelial–mesenchymal transition and migration in TNBC cells and reduced their sensitivity to docetaxel (46). These effects were associated with sensory neuron-derived CGRP and substance P (SP) and activation of PI3K–Akt signaling. In contrast, dynorphin released from sensory neurons activated κ-opioid receptor signaling and partially counteracted these tumor-promoting effects (46). The context-dependent nature of sensory signaling is further illustrated by studies in which pharmacological activation of TRPV1-positive sensory fibers increased intratumoral CD8^+^ T-cell recruitment and IFN-γ responses and reduced metastatic burden in a murine breast cancer model (47). Together, these findings indicate that sensory nerves are not uniformly tumor-promoting, and that their biological effects depend on the neuropeptide released, the responding receptor, and the cellular context.

Beyond breast cancer, studies in other tumor models provide a mechanistic example of how sensory signaling can couple metabolic stress to tumor-cell adaptation. In oral carcinoma models, glucose deprivation induced ROS–c-Jun-dependent NGF production by cancer cells, which stimulated nociceptive neurons to release CGRP (94). Neuron-derived CGRP subsequently promoted cytoprotective autophagy through Rap1-dependent disruption of the mTOR–Raptor complex, thereby supporting tumor-cell survival under nutrient deprivation and reducing the efficacy of nutrient-starvation therapies (94). Although this specific metabolic stress–CGRP–autophagy circuit has not yet been demonstrated in breast cancer, it illustrates a mechanistically plausible route through which sensory neurons can translate metabolic stress into adaptive tumor responses.

Collectively, breast cancer studies establish sensory neuropeptides as regulators of stromal organization, tumor-cell plasticity, immune accessibility, and therapeutic response, while cross-tumor evidence expands the range of metabolic adaptations that may be influenced by sensory signaling.

Across these studies, the neuro–metabolic–immune axis in breast cancer is best understood as a network of convergent interfaces rather than a serial signaling cascade. Lactate and lactylation provide a metabolic–epigenetic interface; adrenergic signaling links neural activity with T-cell metabolic fitness and effector function; and sensory neuropeptides connect tumor-derived cues with stromal remodeling, immune accessibility, and treatment adaptation. These interactions can occur in parallel and may involve distinct cellular compartments within the same tumor. Their relative contribution is therefore likely to vary according to breast cancer subtype, receptor expression, metabolic state, and treatment context. This framework provides a testable basis for identifying spatially defined neural–metabolic–immune niches and for determining which interface represents the dominant therapeutic vulnerability in individual tumors (**Figure 4**).

**Figure 4:**
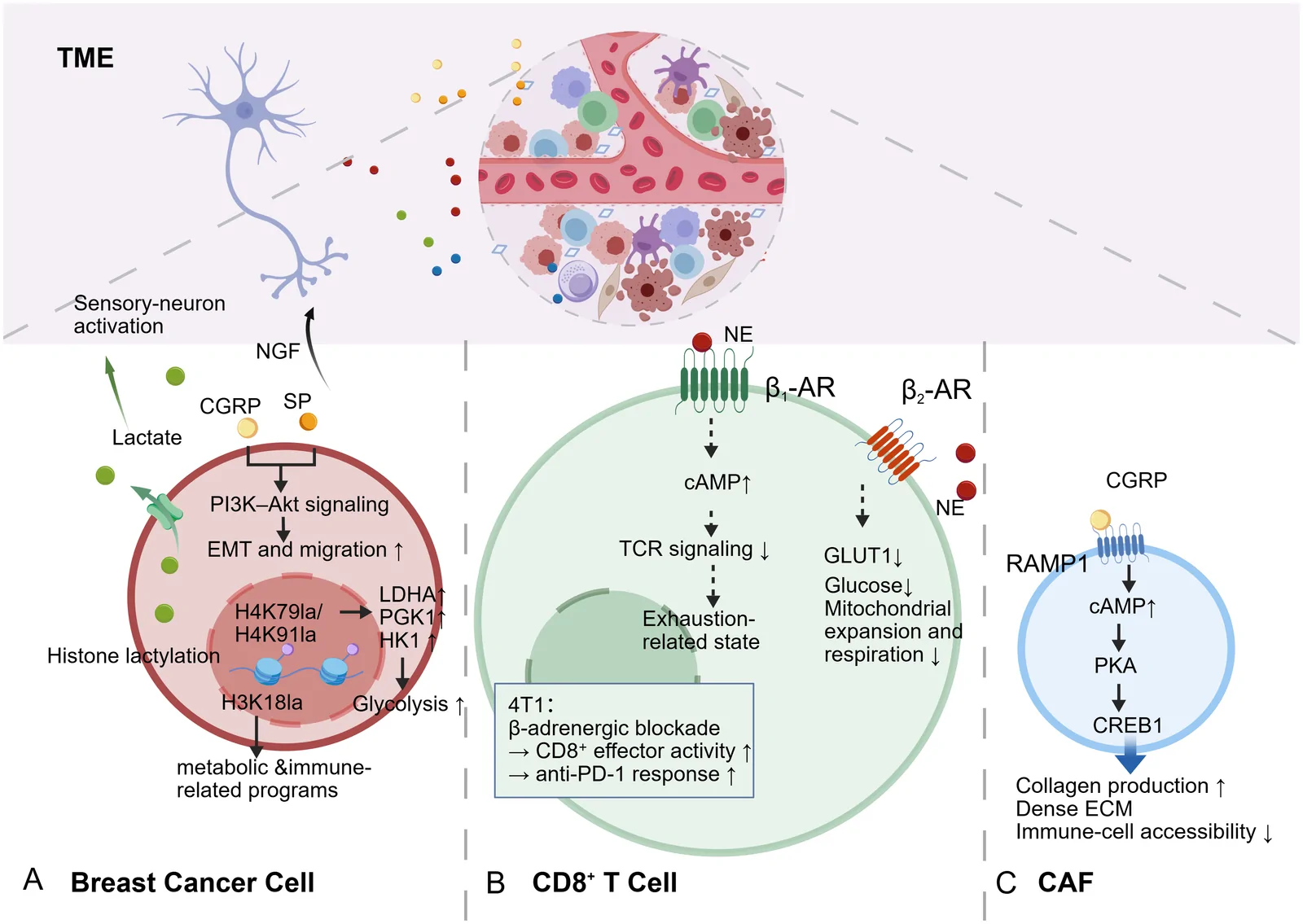
Convergent neuro–metabolic–immune interfaces in breast cancer. **(A)** Breast cancer cells: histone lactylation links lactate accumulation with glycolytic and metabolic–immune transcriptional programs. Sensory neuron-derived CGRP and substance P can promote PI3K–Akt signaling, epithelial–mesenchymal transition, and migration. Lactate released by breast cancer cells can also activate sensory neurons, as demonstrated in bone-metastasis models. **(B)** CD8^+^ T cells: β1-adrenergic signaling can impair T-cell receptor signaling and promote exhaustion-related states, whereas β2-adrenergic signaling can reduce GLUT1 expression, glucose uptake, and activation-associated increases in mitochondrial mass and respiration. These receptor-specific T-cell mechanisms are supported primarily by cross-tumor or non-tumor studies. The inset separately summarizes increased CD8^+^ T-cell effector activity and enhanced responses to anti-PD-1 therapy following β-adrenergic blockade in the 4T1 breast cancer model. **(C)** Cancer-associated fibroblasts: in TNBC, tumor-derived NGF stimulates sensory neurons to release CGRP, which engages the RAMP1-containing receptor complex and activates cAMP–PKA–CREB1 signaling, increasing collagen deposition and limiting immune-cell accessibility. The panels summarize convergent interfaces rather than a single experimentally established causal cascade. Created with BioGDP.com (113).

## 5 Therapeutic implications of neuro-metabolic-immune framework across breast cancer subtypes

Therapeutic translation of the neuro–metabolic–immune framework remains uneven across breast cancer subtypes. Direct neural or neuropeptide-directed approaches, including β-adrenergic blockade and neuropeptide-receptor targeting, coexist with strategies aimed at downstream metabolic or immune vulnerabilities that intersect with neural regulation. Importantly, the supporting evidence spans preclinical models, retrospective clinical associations, and a limited number of prospective studies, and few neural-directed interventions have yet been established as anticancer therapies in breast cancer. Accordingly, we discuss these strategies by molecular subtype while distinguishing direct neural interventions from downstream metabolic or immune targets and considering their current level of clinical maturity (**Tables 2**, **3**).

**Table 2:** Subtype-specific therapeutic opportunities related to neural, neuropeptide, and downstream metabolic signaling in breast cancer.

Molecular subtype	Neural/neuroactive interface	Downstream vulnerability	Representative therapeutic approaches	Current evidence	Key references
Triple-Negative breast cancer (TNBC)	β-Adrenergic signaling; SP–TACR1/NK1R; CGRP–RAMP1	Glutamine dependence; lactate transport and metabolic immunosuppression	β-Blockers; NK1R antagonists; CGRP/RAMP1 blockade; glutaminase inhibition;MCT1/MCT4 inhibition	Level I–III across mechanisms; preclinical to clinical biomarker/observational evidence, with selected strategies in phase II evaluation	(28, 41, 43, 101)
HER2-Positive (HER2+)	β-Adrenergic modulation of HER2-targeted therapy response	FASN-dependent lipid metabolism; glycolytic and lipid-remodeling adaptations associated with resistance	β-Adrenergic modulation; FASN inhibition; PFKFB3 targeting; c-Myc/cPLA2α inhibition	Predominantly Level II–III; mainly preclinical, with early clinical activity reported for FASN inhibition	(105, 107, 112, 116)
HR-positive/HER2-negative	GDNF–RET and NGF-associated neurotrophic signaling	Adipose-derived IGF-1/TXNIP signaling; IL-6-STAT3-SREBF2-HMGCR-dependent cholesterol metabolism associated with CDK4/6 inhibitor resistance	RET inhibition; IGF-1 pathway targeting/TXNIP restoration; HMGCR inhibition ± IL-6 blockade	Predominantly Level III; mainly preclinical/mechanistic with human associative support	(118, 121, 122, 125, 126)

**Table 3:** Selected clinical studies relevant to neural, neuropeptide, and downstream metabolic therapeutic strategies in breast cancer.

Cancer type	Targeted approach	Treatment or exposure	Study design and comparator	Trial identifier	Sample size (n)	Study endpoint and biological response	Clinical findings	Ref.
Interventional studies								
Early-stage BC	Neuroactive signaling: β-adrenergic blockade	Propranolol for 7 preoperative days	Phase II triple-blind biomarker RCT; placebo.	ACTRN12615000889550	60	**Endpoint:** tumor prometastatic and inflammatory gene expression. **Biological response:** EMT and inflammatory signatures decreased, with altered immune profiles.	Completed biomarker study; recurrence and survival benefit untested.	(95)
Stage I–III BC	Neuroactive signaling: β-adrenergic + COX-2 blockade	Propranolol+etodolac for 11 perioperative days	Phase II double-blind biomarker RCT; placebo.	NCT00502684	38	**Endpoint:** tumor EMT, leukocyte-associated and inflammatory and prometastatic signatures. **Biological response:** transcriptional and immune biomarkers modulated; individual drug contributions unresolved.	Biomarker analyses completed; recurrence or survival results not reported. Registry status: Unknown.	(96)
ICI-refractory metastatic/unresectable TNBC	Neuroactive signaling: β-AR blockade	Propranolol+pembrolizumab	Phase II open-label, single-arm; no control.	NCT05741164	Planned 37	**Endpoint:** best objective response within 6 months (irRECIST). **Biological response:** blood and tumor sampling planned; results not reported.	Recruiting; secondary PFS/OS. No efficacy results reported.	(97, 132)
Advanced/metastatic TNBC	Downstream metabolic: glutaminase inhibition	Telaglenastat (CB-839)+paclitaxel	Phase II open-label, 4 single-arm cohorts; no paclitaxel-only control.	NCT03057600	52	**Endpoint:** ORR (RECIST 1.1). **Biological response:** Target engagement or other pharmacodynamic data not reported in posted phase II results.	Completed; ORR 10.0%–53.8% across cohorts; PFS and OS assessed. Added telaglenastat benefit unestablished.	(103)
Trastuzumab-resistant HER2+ metastatic BC	Downstream metabolic: FASN inhibition	Denifanstat (TVB-2640)+trastuzumab+paclitaxel or ET	Phase II open-label, nonrandomized; no control.	NCT03179904	17	**Endpoint:** confirmed ORR (RECIST). **Biological response:** Serum FASN assessed; tumor FASN/pAKT/pS6 analyses pending in cited abstract.	Completed; paclitaxel cohort: 5/15 PRs (ORR 33.3%); median PFS 7.0 months. Early closure; ET cohort inconclusive.	(114, 115)
Observational exposure studies								
BC overall; TNBC subgroup	β-blocker	β-Blocker use at diagnosis	Population cohort+meta-analysis; users vs nonusers.	Not applicable	30,060	**Main outcomes:** BCSS, overall and by subtype. No target-engagement or PD assessment.	No overall BCSS association; TNBC HR 0.66 (95% CI 0.47–0.91). Association only.	(28)
Early BC; TNBC subgroup	TACR1 antagonism	Aprepitant during chemotherapy	Nationwide retrospective cohort; users vs nonusers.	Not applicable	13,811	**Main outcomes:** DDFS and BCSS. No target-engagement or PD assessment.	Associated with better DDFS/BCSS, strongest in TNBC; causality unproven.	(99)
HER2+ advanced BC	β-blocker during anti-HER2 therapy	Pre-existing β-blocker use at anti-HER2 initiation	Pooled observational analysis of 4 RCTs; users vs nonusers; exposure not randomized.	EMILIA, TH3RESA, MARIANNE, CLEOPATRA	2,777	**Main outcomes:** OS and PFS. No target-engagement or PD assessment.	Worse OS association (adjusted HR 1.27, 95% CI 1.04–1.55); no significant PFS association.	(108)

### 5.1 Triple-negative breast cancer

TNBC provides several candidate points for therapeutic intervention within this framework. β-Adrenergic and sensory-neuropeptide pathways represent comparatively direct neural targets, whereas metabolic dependencies involving glutamine utilization and lactate transport constitute downstream vulnerabilities that may intersect with, but are not themselves specific to, neural regulation. Current support for neural-directed strategies derives predominantly from preclinical studies, clinical biomarker studies, and observational analyses, and prospective efficacy evaluation remains limited.

#### 5.1.1 Targeting β-adrenergic signaling

β-adrenergic blockade represents one of the most clinically accessible approaches for targeting stress-associated neural signaling in TNBC. In breast cancer models, β-adrenergic signaling has been linked to tumor-cell invasion, angiogenesis, myeloid-cell accumulation, and impaired antitumor T-cell activity, whereas pharmacological β-blockade can partially reverse several of these effects (20, 21, 23–25, 92). These preclinical findings provide a rationale for repurposing β-blockers as adjuncts to systemic therapy. However, because β-blockers act systemically and can influence circulating catecholamine responses as well as local sympathetic signaling, their effects should not be interpreted exclusively as blockade of intratumoral sympathetic nerves.

Early prospective studies have primarily demonstrated pharmacodynamic and biomarker effects. In a triple-blind, placebo-controlled phase II trial involving 60 patients with operable breast cancer, seven days of preoperative propranolol reduced mesenchymal and proinflammatory transcriptional programs and altered intratumoral immune-cell profiles (95). A separate randomized perioperative study of 38 patients likewise showed that combined propranolol and etodolac treatment modulated prometastatic transcriptional and immune-associated biomarkers (96). Together, these studies demonstrate pharmacodynamic activity of perioperative β-adrenergic blockade in human breast tumors and provide a clinical rationale for efficacy-oriented trials.

Observational evidence has been inconsistent across unselected breast cancer populations. A nationwide cohort study of 30,060 women found no association between β-blocker use and breast cancer-specific survival overall, but a favorable association was observed in the TNBC subgroup; the accompanying meta-analysis similarly identified associations with longer progression- or recurrence-free and breast cancer-specific survival in TNBC (28). These findings support a subtype-specific clinical hypothesis for β-adrenergic blockade in TNBC and provide a rationale for prospective treatment-oriented evaluation.

Building on these proof-of-mechanism studies, β-adrenergic blockade has entered treatment-oriented prospective evaluation. The ongoing phase II trial NCT05741164 is evaluating propranolol in combination with pembrolizumab in patients with checkpoint-inhibitor-refractory metastatic or unresectable TNBC, with objective response as the primary endpoint and progression-free and overall survival among the secondary outcomes. This trial directly tests whether β-adrenergic blockade can enhance or restore responsiveness to immune checkpoint inhibition in advanced TNBC (NCT05741164) (97).

#### 5.1.2 Targeting sensory neuropeptide signaling

The SP–TACR1/NK1R pathway represents a candidate therapeutic target in TNBC. Breast cancer-specific studies have shown that SP–TACR1 signaling can promote metastatic progression through the SP–TACR1–ssRNA–TLR7 pathway, whereas pharmacological TACR1 blockade with aprepitant suppresses tumor growth and metastasis in preclinical models (43). Aprepitant has also enhanced the antitumor activity of doxorubicin while reducing treatment-associated cardiotoxicity in a preclinical TNBC model (98). Among 13,811 women with early breast cancer receiving chemotherapy, aprepitant exposure was associated with improved distant disease-free and breast cancer-specific survival, with the strongest associations observed in TNBC (99). These findings provide a clinical rationale for prospective evaluation of NK1R blockade as a candidate therapeutic strategy in TNBC.

CGRP–RAMP1 signaling represents a second, mechanistically distinct sensory-neuropeptide target in TNBC. Zhang et al. showed that sensory neuron-derived CGRP activates RAMP1-expressing CAFs, increases collagen deposition, and promotes an immune-excluded tumor microenvironment (41). In preclinical TNBC models, the CGRP receptor antagonist rimegepant reduced fibrosis, restored immune-cell infiltration, and enhanced the antitumor activity of anti-PD-1 therapy (41). Rimegepant is clinically approved for the acute and preventive treatment of migraine (100), and these findings provide a preclinical rationale for repurposing CGRP–RAMP1-directed therapy in TNBC.

#### 5.1.3 Targeting glutamine metabolism and lactate transport

Unlike β-adrenergic and sensory-neuropeptide interventions, targeting glutamine metabolism acts on a downstream metabolic dependency rather than directly on neural signaling. Glutamine metabolism represents an important vulnerability in a subset of TNBC, in which glutaminase (GLS) converts glutamine to glutamate to support tricarboxylic acid cycle anaplerosis and redox homeostasis. Telaglenastat (CB-839), a selective GLS inhibitor, preferentially suppressed the growth of glutamine-dependent TNBC models and showed antitumor activity both as a single agent and in combination with paclitaxel (101). More recently, combined inhibition of GLS and the cystine/glutamate antiporter xCT/SLC7A11 depleted intracellular glutathione, increased oxidative stress, and sensitized chemoresistant TNBC models to doxorubicin or cisplatin (102). These findings further support glutamine metabolism as a therapeutic vulnerability in TNBC.

Clinical evaluation of glutaminase inhibition in TNBC has focused primarily on telaglenastat-based combinations. The completed phase II study NCT03057600 evaluated telaglenastat plus paclitaxel in 52 patients with advanced TNBC across four cohorts stratified by ancestry and line of therapy (103). The study evaluated objective response and progression-related outcomes, but did not include a randomized paclitaxel-only comparator. Accordingly, although clinical activity was observed, the specific contribution of telaglenastat cannot be separated from that of paclitaxel.

Lactate transport represents another emerging downstream metabolic–immune target. In 2026, syrosingopine, a dual MCT1/MCT4 inhibitor, was shown to reduce lactate export in TNBC models, decrease tumor-cell PD-L1 expression, restore T-cell effector activity, and enhance NK-cell cytotoxicity. In immunocompetent TNBC models, syrosingopine synergized with anti-PD-1 therapy to suppress tumor growth and prolong survival (104). These findings provide preclinical support for targeting lactate transport to reverse metabolic immune suppression.

### 5.2 HER2-positive breast cancer

In HER2-positive breast cancer, direct neural-targeted therapeutic evidence remains less developed than in TNBC. Current evidence spans β-adrenergic signaling and several downstream metabolic–immune mechanisms associated with resistance to HER2-directed therapy. These strategies differ substantially in their proximity to neural regulation and in their translational maturity.

#### 5.2.1 Adrenergic signaling in HER2-positive breast cancer

β-Adrenergic signaling may influence the response to HER2-targeted therapy, although its effects appear to be highly context dependent. In HER2-overexpressing breast cancer models, catecholamine-driven β2-adrenergic receptor (β2-AR) activation antagonized the antiproliferative effects of trastuzumab through HER2/STAT3 signaling and downstream PI3K–AKT–mTOR activation, whereas propranolol restored trastuzumab sensitivity in resistant models (105). However, tumor ADRB2 expression has shown a different clinical association. In patients with early HER2-positive breast cancer, higher ADRB2 expression was associated with longer disease-free survival and immune-related transcriptional features (106). A subsequent *post-hoc* analysis of 1,282 patients from the NCCTG-N9831 trial similarly showed that ADRB2-high tumors were associated with longer disease-free survival, higher tumor-infiltrating lymphocyte levels, and greater benefit from adjuvant trastuzumab (107). These observations suggest that receptor expression, catecholamine-dependent receptor activation, and pharmacological β-blockade should not be regarded as equivalent measures of β-adrenergic activity.

Clinical studies of β-blocker exposure have likewise yielded inconsistent results. The retrospective clinical analysis accompanying the original β2-AR mechanistic study associated concurrent β-blocker and trastuzumab use with improved progression-free and overall survival (105). In contrast, a pooled analysis of 2,777 patients with advanced HER2-positive breast cancer enrolled in four clinical trials found that pre-existing β-blocker use was associated with shorter overall survival (108), while a subsequent retrospective cohort of 221 trastuzumab-treated patients similarly reported shorter progression-free and overall survival with either selective or non-selective β-blockers (109). β-blockers are also frequently prescribed for cardiovascular comorbidities or treatment-related cardiac risk and can provide cardioprotection during HER2-targeted therapy in selected patients (110). Their clinical associations therefore reflect both oncologic and cardiovascular contexts, and the consequences of β-adrenergic blockade in HER2-positive breast cancer are likely to depend on receptor biology, disease setting, and treatment context.

#### 5.2.2 Targeting FASN-dependent lipid metabolism

Unlike β-adrenergic signaling, fatty acid synthase (FASN) represents a downstream metabolic vulnerability rather than a direct neural target. FASN is closely integrated with HER2 signaling, and pharmacological inhibition of FASN has been shown to reduce HER2 pathway activity and suppress the growth of HER2-positive breast cancer models, including cells with acquired resistance to trastuzumab or lapatinib (111, 112). More recent work has identified the miR-449–FASN axis as an additional mechanism associated with trastuzumab response. Trastuzumab-resistant HER2-positive breast cancer cells showed reduced miR-449 expression and increased FASN levels, whereas restoration of miR-449 or pharmacological FASN inhibition suppressed PI3K–AKT signaling and restored sensitivity to trastuzumab (113). These findings position lipid synthesis as a metabolically tractable component of anti-HER2 resistance.

This metabolic strategy has also entered clinical evaluation. The phase II study NCT03179904 investigated the FASN inhibitor denifanstat (TVB-2640) in combination with trastuzumab and either paclitaxel or endocrine therapy in patients with HER2-positive metastatic breast cancer resistant to HER2-directed therapy (114, 115). Among 16 evaluable patients, 15 received denifanstat, trastuzumab, and paclitaxel; five achieved a partial response, corresponding to an objective response rate of 33.3%, with a median progression-free survival of 7.0 months (114). Enrollment was terminated early for multifactorial reasons, leaving the endocrine-therapy cohort with only one evaluable patient. These early clinical findings support continued investigation of FASN inhibition in anti-HER2-resistant disease, particularly in combination with HER2-directed therapy.

#### 5.2.3 Emerging metabolic–immune vulnerabilities in anti-HER2 resistance

Beyond lipid synthesis, metabolic reprogramming can also interfere with the immune-mediated activity of HER2-targeted therapy. A recent study identified PFKFB3 upregulation as a metabolic feature of trastuzumab-resistant HER2-positive breast cancer (116). Increased PFKFB3 promoted metabolic rewiring and reduced trastuzumab-mediated antibody-dependent cellular cytotoxicity (ADCC), whereas PFKFB3 silencing restored immune-mediated tumor-cell killing in a three-dimensional cancer–immune co-culture system (116). Higher PFKFB3 expression was also associated with poorer survival and incomplete pathological response to trastuzumab in clinical datasets. These findings identify PFKFB3-dependent glycolytic adaptation as a metabolic–immune mechanism of trastuzumab resistance and provide a preclinical rationale for targeting PFKFB3 to restore antibody-mediated cytotoxicity.

Lipid remodeling provides another link between metabolic adaptation and cytokine signaling in anti-HER2 resistance. Wang et al. showed that the histone reader ZMYND8 is upregulated in HER2-positive breast cancers resistant to trastuzumab or trastuzumab plus pertuzumab and promotes c-Myc-dependent expression of cytosolic phospholipase A2α (cPLA2α) (117). The resulting alterations in glycerophospholipid metabolism reduce diacylglycerol-dependent PKC activity and increase IL-27 secretion, thereby promoting resistance to HER2-directed antibodies. Genetic or pharmacological inhibition of c-Myc or cPLA2α restored sensitivity to trastuzumab-based therapy in patient-derived organoids and xenograft models (117). This pathway therefore connects lipid metabolic remodeling with cytokine-dependent resistance and represents a breast cancer-specific downstream metabolic–inflammatory mechanism, with c-Myc and cPLA2α emerging as candidate therapeutic targets for overcoming anti-HER2 resistance.

### 5.3 HR-positive/HER2-negative breast cancer

In HR-positive/HER2-negative breast cancer, therapeutic resistance is shaped by extensive crosstalk between estrogen-receptor signaling, growth-factor pathways, and metabolic adaptation. Neurotrophic signals can sustain estrogen-independent survival programs, whereas adipose-derived metabolic and inflammatory cues further influence endocrine and CDK4/6 inhibitor responses. Together, these processes create multiple therapeutic vulnerabilities across neurotrophic and metabolic pathways.

#### 5.3.1 Neurotrophic signaling in endocrine resistance

GDNF–RET signaling has been implicated in multiple forms of endocrine resistance in ER-positive breast cancer. GDNF-mediated RET activation promotes estrogen-independent ER transcriptional activity through phosphorylation of ERα and downstream signaling that includes mTOR, while RET depletion restores tamoxifen sensitivity in resistant models (118). Subsequent studies extended this association to aromatase inhibitor resistance, where enhanced GDNF–RET signaling was linked to reduced treatment response and increased RET expression in resistant tumors (119).

RET signaling has also been associated with resistance to combined CDK4/6 inhibition and endocrine therapy. High RET expression, particularly when accompanied by elevated GDNF, has been associated with unfavorable outcomes and endocrine-resistant transcriptional programs in ER-positive breast cancer (120). RET was subsequently found to be upregulated in ER-positive models resistant to combined CDK4/6 inhibition and fulvestrant (121).

NGF signaling has also been linked to cell survival and endocrine response in ER-positive breast cancer. BEX2 identifies a subset of ER-positive tumors with distinct responses to tamoxifen and participates in NGF–p75NTR–NF-κB signaling that regulates apoptosis in breast cancer cells (122). BEX2 expression was required for NGF-mediated protection from apoptosis and also modified the antiproliferative response to tamoxifen (122). These findings position NGF-p75NTR-BEX2 signaling as a potential contributor to endocrine response in ER-positive breast cancer.

#### 5.3.2 Adipose-associated metabolic signaling

Adipose tissue can influence endocrine response in HR-positive breast cancer through local inflammatory and hormonal changes. In human breast adipose tissue, macrophage-rich crown-like structures are associated with local inflammation and increased aromatase expression and activity (123). Consistent with a clinical influence of adiposity on endocrine-treated disease, obesity has been associated with a higher risk of recurrence in postmenopausal patients with HR-positive breast cancer receiving adjuvant aromatase inhibitors (124). These findings link the breast adipose microenvironment with local estrogen production and endocrine treatment outcomes.

Mechanistic studies have further identified adipocyte-derived signals that can reduce endocrine sensitivity. Caruso et al. showed that adipocyte-derived IGF-1 suppresses TXNIP and promotes resistance to tamoxifen and aromatase inhibition in ER-positive breast cancer models (125). Targeting IGF-1 signaling or restoring TXNIP attenuated these effects, and the findings were validated using human mammary adipocytes and patient-derived organoids. An IGF-1high/TXNIPlow signature was also associated with poorer outcomes in patients receiving endocrine therapy (125). These results identify the IGF-1-TXNIP pathway as a potential mediator of adipocyte-associated endocrine resistance.

More recently, Huang et al. extended adipocyte-associated resistance to CDK4/6 inhibition in ER-positive breast cancer. Cancer-associated adipocyte-derived IL-6 activated JAK–STAT3 signaling and induced SREBF2, which promoted resistance through HMGCR-dependent cholesterol metabolism and CDKN2C-mediated cell-cycle progression (126). Pharmacological inhibition of HMGCR with simvastatin, alone or combined with IL-6 blockade, restored CDK4/6 inhibitor sensitivity across complementary preclinical models (126). These findings provide a preclinical rationale for targeting adipocyte-driven cholesterol metabolism and inflammatory signaling in CDK4/6 inhibitor-resistant disease and represent Level III evidence for a downstream adipose–metabolic–inflammatory mechanism within the broader framework.

### 5.4 Translational challenges and future directions

A major challenge in translating neuro–metabolic–immune interactions into breast cancer therapy is the uneven maturity of the supporting evidence. Neural and neuropeptide pathways are often defined in breast cancer models, whereas downstream metabolic and immune mechanisms may derive from different experimental systems or other malignancies. Thus, biological plausibility does not necessarily indicate that a complete neuro-metabolic-immune circuit operates as a continuous causal pathway in human breast tumors. Future studies should prioritize validation of linked mechanisms within the same breast cancer model and, where possible, in paired human tissue and treatment-response datasets.

A second challenge is the pronounced spatial and cellular heterogeneity of neural signaling. Neurotransmitter and neuropeptide effects depend on receptor subtype, responding cell population, ligand availability, and treatment context, limiting the interpretability of bulk expression measurements. Spatial transcriptomics, multiplex imaging, and single-cell approaches will therefore be important for identifying nerve-associated tumor, stromal, and immune niches and for developing biomarkers of neuromodulatory responsiveness.

Therapeutic specificity also remains a major concern. β-Blockers, NK1R antagonists, and CGRP-pathway inhibitors act systemically and may affect cardiovascular, neurological, and immune physiology beyond the tumor microenvironment; their effects therefore cannot be assumed to reflect selective inhibition of intratumoral neural signaling. Future development should consider receptor selectivity, tissue distribution, dosing, and strategies that restrict exposure to the relevant tumor or stromal compartments.

Clinical translation should therefore proceed through biomarker-informed and mechanistically designed studies rather than efficacy testing alone. Early trials should distinguish direct neural interventions from downstream metabolic or immune therapies, confirm target engagement, and account for breast cancer subtype, treatment setting, comorbid indications, and concomitant medications. Window-of-opportunity studies and paired pre- and post-treatment sampling may be particularly useful for determining whether neural modulation alters tumor proliferation, metabolic state, immune composition, or response to standard therapy and for identifying biomarkers that can guide subsequent efficacy-oriented trials.

## 6 Conclusion

Growing evidence has established neural signaling as an important component of the breast tumor microenvironment. Sympathetic, cholinergic, and sensory pathways can influence cancer cells, stromal populations, and immune cells through neurotransmitters, neuropeptides, and neurotrophic factors. At the same time, metabolic programs involving glucose, lipids, amino acids, and lactate shape tumor behavior and immune function. Increasingly, these processes are being found to intersect, with neural signals influencing selected metabolic and stromal programs and metabolic changes, in turn, modifying immune and cellular states within the tumor microenvironment.

The neuro–metabolic–immune axis, as an integrative framework, provides a useful lens for organizing the converging neural, metabolic, and immune mechanisms in breast cancer. However, the strength of evidence varies considerably across components, and the axis should be viewed as a testable model rather than a fully established pathway. Adrenergic signaling, sensory neuropeptides, neurotrophic factors, and lactate-associated metabolic and epigenetic regulation represent several important points of convergence. Some of these interactions have been demonstrated directly in breast cancer, whereas others remain supported mainly by preclinical or cross-tumor evidence. Defining how these pathways operate together in human tumors will be essential for determining which components are biologically dominant and clinically actionable.

Therapeutically, neural and neuropeptide pathways offer opportunities for direct neuromodulation, while metabolic and immune pathways may provide downstream vulnerabilities that can be targeted in selected disease contexts. β-Adrenergic signaling, NK1R/TACR1, CGRP–RAMP1 signaling, and related neuroactive pathways are at different stages of translational development, and their therapeutic effects are likely to depend on receptor and cell-type specificity, disease subtype, and treatment context. Future studies integrating spatial profiling, single-cell analysis, longitudinal sampling, and biomarker-guided clinical investigation will be needed to define functional neural–tumor–immune niches and to identify patients most likely to benefit from these approaches. Clarifying where neural, metabolic, and immune regulation converge—and where they remain biologically distinct—will be essential for translating selected components of the axis into rational strategies for overcoming treatment resistance in breast cancer.

## Glossary

ACh: Acetylcholine
ADCC: Antibody-dependent cellular cytotoxicity
ADRB1: Adrenergic receptor β1
ADRB2: Adrenergic receptor β2
APC: Antigen-presenting cell
ATGL: Adipose triglyceride lipase
BC: Breast cancer
BCSS: Breast cancer-specific survival
BDNF: Brain-derived neurotrophic factor
BEX2: Brain expressed X-linked 2
CAF: Cancer-associated fibroblast
cAMP: Cyclic adenosine monophosphate
CGRP: Calcitonin gene-related peptide
CHRNA7: Cholinergic receptor nicotinic alpha 7 subunit
CHRNA9: Cholinergic receptor nicotinic alpha 9 subunit
cPLA2α: Cytosolic phospholipase A2 alpha
CREB1: cAMP response element-binding protein 1
CSC: Cancer stem cell
CTL: Cytotoxic T lymphocyte
CTLA-4: Cytotoxic T-lymphocyte-associated protein 4
DDFS: Distant disease-free survival
ECM: Extracellular matrix
EMT: Epithelial–mesenchymal transition
ER: Estrogen receptor
ERα: Estrogen receptor alpha
ERK: Extracellular signal-regulated kinase
ET: Endocrine therapy
FAO: Fatty acid oxidation
FASN: Fatty acid synthase
FDG: Fluorodeoxyglucose
GABA: γ-Aminobutyric acid
GDNF: Glial cell line-derived neurotrophic factor
GLS: Glutaminase
GLUT1: Glucose transporter 1
H3K18la: Histone H3 lysine 18 lactylation
H4K79la: Histone H4 lysine 79 lactylation
H4K91la: Histone H4 lysine 91 lactylation
HER2: Human epidermal growth factor receptor 2
HIF-1α: Hypoxia-inducible factor 1 alpha
HK2: Hexokinase 2
HR: Hormone receptor
ICI: Immune checkpoint inhibitor
IFN-γ: Interferon gamma
IGF-1: Insulin-like growth factor 1
ILC2: Type 2 innate lymphoid cell
LDHA: Lactate dehydrogenase A
mAChR: Muscarinic acetylcholine receptor
MCT4: Monocarboxylate transporter 4
MDSC: Myeloid-derived suppressor cell
NE: Norepinephrine
nAChR: Nicotinic acetylcholine receptor
NF-κB: Nuclear factor kappa B
NGF: Nerve growth factor
NK: Natural killer
NK1R: Neurokinin 1 receptor
NMDAR: N-Methyl-D-aspartate receptor
NPG: Neuron-projection-guidance
ORR: Objective response rate
OS: Overall survival
p75NTR: p75 neurotrophin receptor
PD: Pharmacodynamic
PD-1: Programmed cell death protein 1
PD-L1: Programmed death-ligand 1
PFKFB3: 6-Phosphofructo-2-kinase/fructose-2,6-bisphosphatase 3
PFS: Progression-free survival
PI3K: Phosphoinositide 3-kinase
PKA: Protein kinase A
PNI: Perineural invasion
PPARγ: Peroxisome proliferator-activated receptor gamma
RAMP1: Receptor activity-modifying protein 1
RCT: Randomized controlled trial
Ref-1: Redox effector factor 1
RET: Rearranged during transfection receptor tyrosine kinase
ROS: Reactive oxygen species
SC: Schwann cell
SERT: Serotonin transporter
SP: Substance P
ssRNA: Single-stranded RNA
STAT3: Signal transducer and activator of transcription 3
TACR1: Tachykinin receptor 1
TAM: Tumor-associated macrophage
TH: Tyrosine hydroxylase
TIL: Tumor-infiltrating lymphocyte
TLR7: Toll-like receptor 7
TME: Tumor microenvironment
TNBC: Triple-negative breast cancer
TrkA: Tropomyosin receptor kinase A
TrkB: Tropomyosin receptor kinase B
TRPV1: Transient receptor potential vanilloid 1
TXNIP: Thioredoxin-interacting protein
USP22: Ubiquitin-specific peptidase 22
USP28: Ubiquitin-specific peptidase 28
VEGF: Vascular endothelial growth factor
Xct/SLC7A11: Cystine/glutamate antiporter/solute carrier family 7 member 11
β1-AR: β1-Adrenergic receptor
β2-AR: β2-Adrenergic receptor
